# Low toxicity and favorable clinical and quality of life impact after non-myeloablative autologous hematopoietic stem cell transplant in Crohn’s disease

**DOI:** 10.1186/s13104-017-2824-1

**Published:** 2017-10-06

**Authors:** Milton Artur Ruiz, Roberto Luiz Kaiser, Luiz Gustavo de Quadros, Lilian Piron-Ruiz, Tatiana Peña-Arciniegas, Mikaell Alexandre Gouvea Faria, Rubens Camargo Siqueira, Flavio Fontes Pirozzi, Fernanda Soubhia Liedtke Kaiser, Richard K. Burt

**Affiliations:** 1 Associação Portuguesa de Beneficência, St. Catarina Nucci Parise 760-SJ Rio Preto, Sao Jose Do Rio Preto, SP 15090 470 Brazil; 2 Faculdade de Medicina da Universidade de São Paulo, São Paulo, SP Brazil; 3 Departamento de Genética Unesp/Ibilce Sao Jose do Rio Preto, Sao Jose Do Rio Preto, SP Brazil; 4 Kaiser Clinica, Sao Jose Do Rio Preto, SP Brazil; 5 Faculdade de Medicina da Universidade de São Paulo, Ribeirão Preto, São Paulo, SP Brazil; 6 Division of Immunotherapy, Northwestern University Feinberg School of Medicine, Chicago, IL USA

## Abstract

**Objective:**

The incidence of adverse events in myeloablative transplant protocols is high in refractory Crohn’s disease; this study used low doses of cyclophosphamide. Fourteen patients were submitted to non-myeloablative autologous hematopoietic stem cell transplantation.

**Results:**

The average number of days of anemia (hemoglobin < 10 g/dL) was 5.4 ± 4.2 and 14 ± 2.4 in the mobilization and conditioning phases, respectively. The mean number of days of neutropenia (neutrophils < 0.5 × 10^9^/L) in the mobilization phase was 1.7 ± 1.5 while it was 7.6 ± 1.4 in the conditioning phase. When comparing the conditioning and mobilization phases, there was an increased number days of leukopenia (white blood cells < 1.0 × 10^9^/L), lymphocytopenia (lymphocytes < 0.5 × 10^9^/L) and thrombocytopenia (platelets < 25 × 10^9^/L). Crohn’s Disease Activity Index values before the transplant ranged from 155 to 450.5 (mean 281.2 ± 79.0) and at 30 days after the procedures they ranged from 45.4 to 177 (mean 95.8 ± 35.4). Moreover, the procedure improved in overall quality of life of patients. Non-myeloablative autologous hematopoietic stem cell transplantation with lower doses of cyclophosphamide leads to lower rates of hematological toxicity and adverse events compared to protocols described in the literature.

*Trial registration* NCT 03000296: Date 9 December 2016

**Electronic supplementary material:**

The online version of this article (doi:10.1186/s13104-017-2824-1) contains supplementary material, which is available to authorized users.

## Introduction

Crohn’s disease is a chronic, idiopathic condition with high prevalence and annual incidence from 5.0 to 20.2 per 100,000 person-years [1, 2]. Approximately 80% of all patients require intestinal surgery including permanent stomas, and many develop tumors [3–5]. Therapy includes anti-inflammatory drugs, steroids, immunosuppressants and biological agents [6, 7]. Patients with refractory disease should undergo non-conventional therapies such as autologous hematopoietic stem cell transplantation (aHSCT) [8].

However, transplantation has limited effectiveness with several possible complications, including reduced organ function and infections, and it is not considered standard treatment for Crohn’s disease [9].

Nearly all patients in the Autologous Stem Cell Transplantation International Crohn’s Disease (ASTIC) trial experienced non-serious adverse events [10].

Most complications were related to myeloablation during the conditioning phase including irreversible bone marrow failure, which might be attributed to the high doses of cyclophosphamide used for mobilization [11]. Consequently, non-myeloablative aHSCT regimens have been explored.

This study describes the preliminary results of 14 patients in a larger study. The patients were diagnosed with refractory Crohn’s disease and submitted to a non-myeloablative regimen with low doses of cyclophosphamide.

## Main text

### Methods

This study describes the baseline conditions, treatment protocols, and clinical outcomes of 14 patients diagnosed with Crohn’s disease. All patients underwent aHSCT using low doses of cyclophosphamide during mobilization.

#### Patients

Inclusion criteria were Crohn’s Disease Activity Index (CDAI) greater than 150, intestinal lesions detected by colonoscopy or capsule endoscopy, disease refractory to treatment, a history of adverse reactions to two biologic agents, conditions preventing additional surgical procedures and risk of a permanent stoma and rectal amputation.

All patients with significant comorbidities, including Hodgkin’s lymphoma, chronic myeloid leukemia and liver disease, were excluded, as were patients in remission, and with coexisting psychiatric disorders, infectious diseases, fistulae, ulcerative colitis, or neoplastic disorders.

#### Mobilization phase

To safely perform the transplant, our goal was a CD34^+^ count above 3.5 × 10^6^/kg. Patients were administered a single dose of cyclophosphamide (60 mg/kg-Baxter) with Sodium-2 Mercaptoethanesulphonate ((mesna)—60 mg/kg—Blau Farmaceutica) as prophylaxis. Five days later, granulocyte colony-stimulating factor (G-CSF-10 mcg/kg/day—Amgen) was administered and maintained until after peripheral hematopoietic stem cell (PHSC) harvesting. Patients with neutrophil levels below 0.5 × 10^9^/L received intravenous cefepime (1 g b.i.d.-Bristol) until the end of harvesting. The PHSC were cryopreserved at − 87 °C without selection or manipulation. Leukopheresis sessions were daily until the CD34^+^ cell concentration in the peripheral blood was above 8/mL. At the end of harvesting, patients refrained from major physical activities for 7–10 days until the conditioning phase.

#### Conditioning phase

Conditioning consisted of cyclophosphamide (50 mg/kg for 4 days) and total dose of 6.5 mg/kg rabbit antithymocyte globulin (rATG—Genzyme) administered daily for 4 days prior to the PHSC infusion. Methyl prednisolone (Pfizer—500 mg/day) was also administered prior to the rATG to reduce the risk of adverse reactions. Additionally, mesna (50 mg/kg for 4 days) was administered to reduce the risk of toxicity. In cases of neutropenia (< 0.5 × 10^9^/L) occurring between days 1 and 5, 10 μg/kg/day of G-CSF was administered after the infusion of PHSC and maintained until the absolute neutrophil and platelet counts reached 0.5 × 10^9^/L and 20 × 10^9^/L, respectively.

#### Supportive care

Sulfamethoxazole (400 mg PO—Neoquimica) and trimethoprim (80 mg PO—Neoquimica) were administered three times/day for prophylaxis against *Pneumocystis jiroveci* during the 4 days prior to the PHSC infusion. Other medications prophylactically administered included ciprofloxacin (500 mg PO b.i.d.—Eurofarma), metronidazole (250 mg PO t.i.d.—Eurofarma), acyclovir (200 mg PO t.i.d.—Teuto) and fluconazole (150 mg PO b.i.d.). Cefepime (1 g IV b.i.d.) was administered if neutropenia occurred. Targocid (400 mg/day IV—Sanofi) was administered if fever reached 38.2 °C and replaced with meronen (2 g IV b.i.d.) if the fever continued. Further episodes of fever were treated with polymyxin B (Eurofarma/Quimiica Haller). Blood samples were collected from the central venous catheter for culturing. Transfusion of red blood cell concentrates was determined by hematocrit values below 25% and platelet transfusions were indicated when counts were below 20 × 10^9^/L. Blood components were deleucotized by irradiation prior to transfusion.

#### Outcomes

Clinical evaluations were performed before and for up to 30 days after transplantation. This assessment included the CDAI [12], Crohn’s Disease Endoscopic Index of Severity (CDEIS) [13], Simple Endoscopic Score for Crohn’s Disease (SES-CD) [14], Crohn’s Severity Index (CSI), Harvey–Bradshaw index (HBI) [15], and Rutgeerts Score to evaluate the anastomosis after ileocolic resection [16]. The Bristol stool scale was used to categorize frequency and consistency of patients’ stools [17]. Toxicity was evaluated according to the National Cancer Institute Common Criteria for toxicity [18], and quality of life (QoL) according to the Short Form-36 (SF-36) [19].

#### Statistical analysis

Data analysis was descriptive (means, frequencies and percentages) with most results being reported for individual patients. All analyses were performed using the R language [20] and the ggplot2 [21], rmarkdown [22] packages.

#### Ethical considerations

This study registered was as a trial (NCT 03000296: Date 9 December 2016) and reviewed and approved by the Ethics Review Board for Research involving Human Beings of the Hospital da Associacao Portuguesa de Beneficencia Sao Jose do Rio Preto and all patients provided written informed consent.

### Results

Seven male and seven female patients were evaluated with a mean age of 35.9 years (range 24–50 years). Details about the patient demographics and clinical manifestations prior to aHSCT are shown in Additional file 1.

#### Patient outcomes

Eleven patients were classified using the CDEIS and SES-CD scores while seven were classified using Rutgeerts scale. Two patients did not follow any of the above classifications given a history of extensive resection surgeries, although they were diagnosed as having Crohn’s disease through entero-resonance and/or capsule endoscopy. Table 1 shows the classification of patients according to the different scores.

**Table 1:** Scores prior to autologous hematopoietic stem cell transplantation

Patient#	HBI	CSI	Montreal	CDEIS	SES-CD	Rutgeerts	CDAI
1	37	30	A2 L4 B2	17	18	i4	450.5
2	18	22	A3 L3 B2	6	8	–	247
3	22	20	A1 L3 B1	11	14	–	306
4	48	35	A1 L1 B1	–	–	–	427
5	15	28	A2 L3 B2	1	4	–	240
6	14	23	A2 L3 B1	7	6	i4	250
7	15	32	A2 L3 B1p	15	22	–	261
8	14	20	A2 L3 B2	14	14	i4	273.7
9	21	31	A3 L3 B3p	–	–	–	308
10	22	28	A3 L3 B3p	8	7	i4	260
11	16	16	A2 L3 B2	10	13	i4	284.8
12	13	36	A2 L3 B2P	36	24	i4	287.5
13	14	34	A2 L3 B2	34	11	i4	186
14	11	17	A1 L3 B2P	–	–	–	155
Mean ± SD	20 ± 10.3	26.5 ± 6.8		14.5 ± 11.1	12.8 ± 6.5		281.2

*HBI* Harvey–Bradshaw index; *CSI* Crohn’s severity index; *CDEIS* Crohn’s disease endoscopic index of severity; *SES-CD* simple endoscopic score for Crohn’s disease; *CDAI* Crohn’s disease activity index; *SD* standard deviation

CDAI scores 30 days after the procedure ranged from 45.4 to 177.0 (mean 95.8 ± 35.4). Thirteen patients presented CDAI scores below 150 within 30 days after the transplant.

#### Crohn’s disease kinetics

The mean number of cells collected during leukapheresis was 13.4 × 10^6^ ± 9.5 × 10^6^/kg (range 4.3–36.7 × 10^6^) with no adverse reactions being reported, which demonstrated that all patients presented good mobilization. After the PHSC infusion, all patients reported taste modifications, abdominal discomfort and dark urine at the first urination. Patients took an average of 9.8 days to obtain neutrophil levels greater than 0.5 × 10^9^/L. A similar period was observed to reach platelet counts above 25 × 10^9^/L.

Data on Crohn’s disease kinetics during mobilization and conditioning are shown in Table 2.

**Table 2:** Crohn’s disease kinetics during mobilization and conditioning phases

Patient#	CD34^+^ harvested and infused (× 10^6^)	Number of apheresis sessions	Neutrophil graft day	Platelet graft day	Number of units of RBCs (mobilization/ conditioning)	Number of units of platelets (mobilization/ conditioning)
1	4.59	2	7	7	0/0	0/0
2	8.52	2	9	9	0/0	0/0
3	7.4	2	9	9	0/2	0/0
4	5.07	1	10	10	0/1	0/0
5	4.3	1	12	12	0/2	0/1
6	7.7	2	10	10	0/2	0/2
7	10.2	2	10	10	0/7	0/2
8	8.1	1	10	10	0/2	0/4
9	22	1	9	9	0/2	0/1
10	18.67	1	11	11	0/2	0/5
11	36.65	2	11	11	2/11	0/3
12	22.3	1	9	9	1/1	0/0
13	19.7	1	10	10	1/2	0/3
14	20	1	10	10	0/4	0/3

#### Complications

All patients presented with diarrhea during the mobilization phase (mean days 13.6 ± 5.1). The frequency increased when the patient had neutropenia during the conditioning phase (average days of diarrhea 17.2 ± 3.7). Digestive symptoms improved after the mobilization phase with a progressive reduction in diarrheal episodes until symptoms disappeared.

An additional table lists complications following HSCT (see Additional file 2) and hematological abnormalities during conditioning and mobilization are shown in Additional file 3.

#### Quality of life

**Fig. 1:**
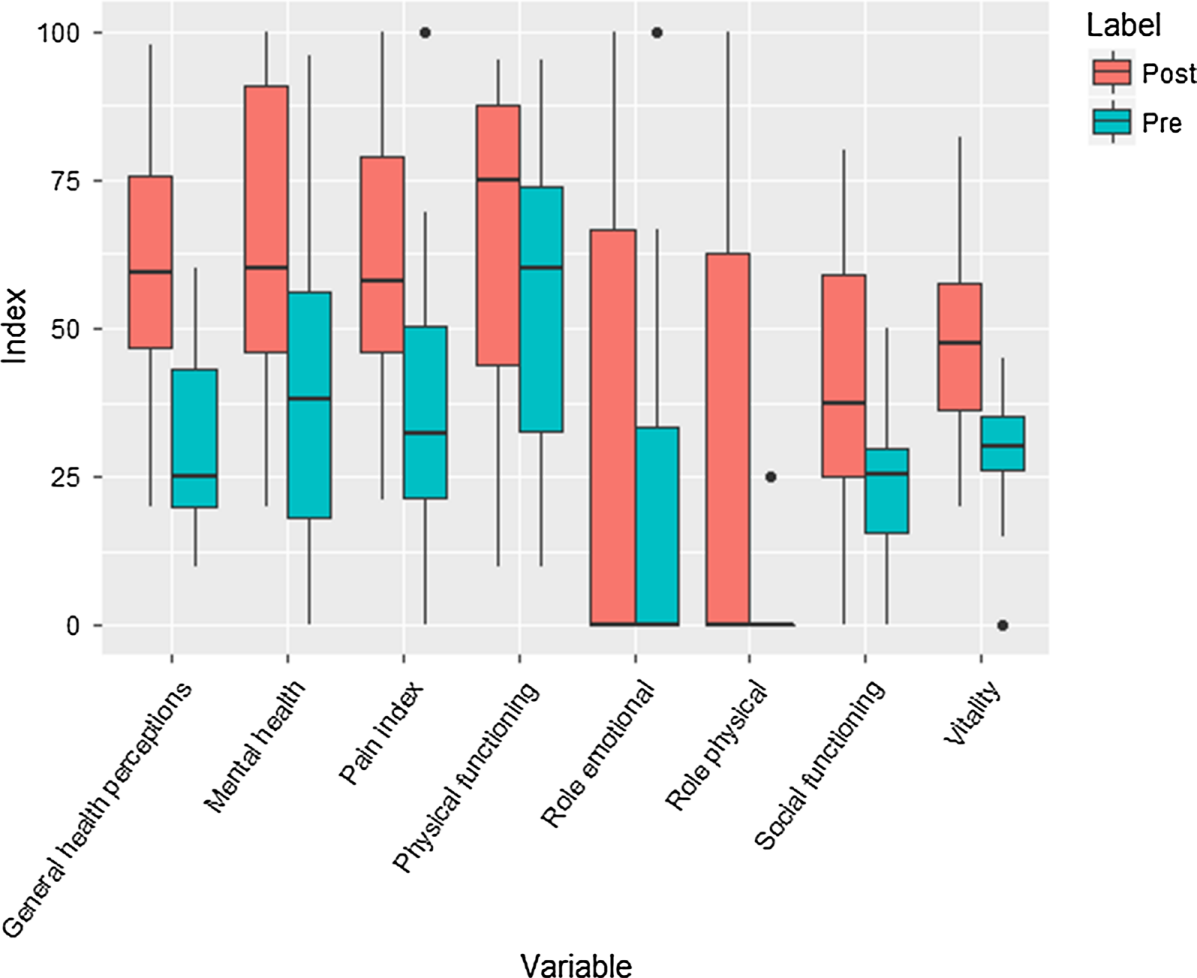
Quality of life, variable means before and after aHSCT

An overall improvement in the different SF-36 domains was observed after aHSCT (Fig. 1).

### Discussion

This study describes a non-myeloablative regimen for aHSCT with low doses of cyclophosphamide during mobilization. Clinical outcomes 30 days after aHSCT were better than published results. Prior to the procedure, all patients presented CDAIs > 150 whereas 30 days after aHSCT, 13 patients were in remission with CDAIs < 150. This non-myeloablative protocol resulted in low toxicity with only four patients having complications directly related to the transplant.

The protocol of published studies consisted of a conditioning regimen with anti-lymphocyte globulin or total body irradiation followed by the infusion of PHSC collected by apheresis [23]. Early ablative regimens presented high toxicity without improving clinical outcomes compared to the standard Crohn’s disease treatment [8]. Contrary to the current study with substantial improvements within 1 month, the first aHSCT case series involving four patients with Crohn’s disease resulted in an improvement in CDAI scores 3 months after procedure [8] with three of the four patients presenting unexpected adverse events (perianal abscess after mobilization, pleural and pericardial effusions, and macro-hematuria). In a subsequent case series involving 12 patients, nearly all presented with hematological abnormalities, neutropenic fever, disease-related fever, diarrhea, anorexia, nausea and vomiting [24]. In addition, the median days for neutrophil and platelet engraftment were 9.5 (range 8–11) and 9 (range 9–18), respectively, compared to 1.5 (range 0–4) and 0.0 (range 0–1) days in the current study.

Non-myeloablative regimens have been designed to reduce the time of neutropenia and immunosuppression [11]. Cyclophosphamide was previously used at doses of 4 g/m^2^ [26], 2 g/m^2^ for 2 days [10, 27] and 2 g/m^2^ for 4 days [11] during mobilization. In contrast, this study used 50–60 mg/kg/day (2 g/m^2^) of cyclophosphamide administered over 1 day. The better outcomes reported here might be explained by the lower doses of cyclophosphamide during mobilization.

In one study involving 26 patients, mobilization, with a median duration of 5 days (range 2–7), resulted in the following complications: febrile neutropenia (16 patients), bacteremia (one patient) and septic shock (two patients). Other complications included acute renal failure in one patient with septic shock, pharmacodermia following the use of vancomycin, and anemia. Approximately 80% of all patients required transfusions after their hemoglobin levels dropped below 12 g/dL and only 21 patients proceeded to the conditioning and transplantation phases. The mean time before starting conditioning after leucopheresis was 53 days (range 14–458), and neutropenia and thrombocytopenia lasted for a median of 11 (range 7–16) and 4 days (range 2–8), respectively. During the conditioning phase, 20 patients (95%) presented febrile neutropenia, one presented with septic shock, perianal lesions worsened in three patients, and all patients required transfusions. This case series also described non-infectious complications in six cases (29%), including reactions to the use of rATG with severe hypotension, and 12 (57%) patients presenting with mucositis (Berman grades I–II) [25]. Conversely, during the mobilization phase of the current study, febrile neutropenia was observed in one patient (7%), no patient presented bacteremia or septic shock and only three patients (21%) required blood transfusions. In the conditioning phase, four patients (29%) presented with febrile neutropenia with confirmed evidence of bacteremia, with 13 (93%) patients requiring blood transfusions. In contrast to our protocol, the ASTIC study [10] used twice the dose of cyclophosphamide during the mobilization phase (4 g/m^2^). Our group has previously suggested that this dosage was high for mobilization, ultimately leading to toxicity [26]. In fact, the ASTIC study reported frequent serious adverse events (79 in 19 patients) with nearly all patients experiencing non-serious adverse events. However, no serious adverse events were reported, while non-serious adverse events were observed in all patients. In the study by Burt [11], 11 patients (46%) presented with infectious complications during hospitalization, while in this study only three (21%) patients presented with infectious complications. Besides the different dosage, the higher rates of complications in the ASTIC trial might be explained because it was multicentric, where compliance with the protocol might vary across sites.

As far as we know, this study is the first to evaluate early QoL following aHSCT. Previous studies have demonstrated poor functional outcomes in patients with severe active Crohn’s disease including psychosocial dysfunction, addiction to narcotics, decreased productivity and reduced QoL scores [27]. However, remission in this condition is associated with improved function including QoL [28]. This is consistent with findings of this study where there was an overall improvement in all SF-36 domain scores after remission.

The current study demonstrates lower hematological toxicity with fewer infectious complications and adverse events following aHSCT compared to previous studies. We therefore recommend that future studies should consider the use of lower doses of cyclophosphamide in mobilization regimens.

## Limitations

This study has limitations frequently associated with case series. First, there was no control group, which precludes comparisons with traditional, ablative treatments. Second, the sample size is small, not allowing for the evaluation of risk factors. Last, this is a single-center study, and so the effect of multiple centers on outcomes cannot be determined.

## Additional files

**Additional file 1.** Sociodemographic and clinical characteristics.

**Additional file 2.** Complications following autologous hematopoietic stem cell transplantation.

**Additional file 3.** Hematological abnormalities during autologous hematopoietic stem cell transplantation.

## Abbreviations

mesna: Sodium-2-Mercaptoethanesulphonate
PHSC: peripheral hematopoietic stem cells
AGA: American Gastroenterological Association’s
ATG: antithymocyte globulin
aHSCT: autologous hematopoietic stem cell transplantation
ASTIC: Autologous Stem Cell Transplantation International Crohn’s Disease
CDAI: Crohn’s disease activity index
CDEIS: Crohn’s disease endoscopic index of severity
CSI: Crohn’s severity index
G-CSF: granulocyte colony-stimulating factor
HBI: Harvey–Bradshaw index
PO: per os
SF-36: Short Form-36
SES-CD: Simple Endoscopic Score for Crohn’s Disease
QoL: quality of life
