# Supplementary material for: Low toxicity and favorable clinical and quality of life impact after non-myeloablative autologous hematopoietic stem cell transplant in Crohn’s disease
Source: BMC Res Notes. 2017 Oct 6;10:495. doi: 10.1186/s13104-017-2824-1 (PMC5639601; doi:10.1186/s13104-017-2824-1)
Supplement: Supplementary file 1 — Additional file 1. Sociodemographic and clinical characteristics. [file 13104_2017_2824_MOESM1_ESM.docx]

**Sociodemographic and clinical characteristics**

| Patient # | Symptoms duration prior to diagnosis (years) | Prior surgery | Narcotics | Perianal fistula | Prior treatment complications |
| --- | --- | --- | --- | --- | --- |
|  |  |  |  |  |  |
| 1 | <1 | Resection terminal ileum, caecum | No | None | None |
| 2 | 15 | Resection ileum colon terminal | Yes | None | None |
| 3 | 1 | None | Yes | None | None |
| 4 | 19 | None | No | None | None |
| 5 | 4 | Colectomy right and segmented | No | None | None |
| 6 | 3 | Colectomy right | No | Perianal fistula | None |
| 7 | 4 | Anal fistulectomy | Yes | None | None |
| 8 | 2 | Resection ileum anal fistula terminal | Yes | Perianal fistula | Allergy Infliximab bronchospasm |
| 9 | 14 | Total colectomy, fistulectomy (n = 3) | No | Perianal fistula | None |
| 10 | 5 | Resected terminal ileum colon, right sphinterectomy | No | None | Chickenpox suspended Infliximab |
| 11 | 5 | Resection of terminal ileum, caecum | Yes | Perianal fistula | None |
| 12 | 1 | Fistulectomy | No | None | None |
| 13 | 1 | Total colectomy, fistulectomy | Yes | Colostomy | Allergy Infliximab 3^rd^ application |
| 14 | 1 | Fistulectomy perianal dilation (n = 3) | No | Anal obstruction | Allergy Infliximab Severe itching |

* Patient 13 was the only patient with colostomy and severe dermatitis, which improved with treatment after HSCT.
