# Supplementary material for: Low toxicity and favorable clinical and quality of life impact after non-myeloablative autologous hematopoietic stem cell transplant in Crohn’s disease
Source: BMC Res Notes. 2017 Oct 6;10:495. doi: 10.1186/s13104-017-2824-1 (PMC5639601; doi:10.1186/s13104-017-2824-1)
Supplement: Supplementary file 2 — Additional file 2. Complications following autologous hematopoietic stem cell transplantation. [file 13104_2017_2824_MOESM2_ESM.docx]

**Complications following autologous hematopoietic stem cell transplantation**

| Patient | Days  of  fever | Infection  (Mob/Cond) | Days with  diarrhea  (Mob/Cond) | Days with abdominal  Pain (Mob/Cond) | Other complications |
| --- | --- | --- | --- | --- | --- |
|  |  |  |  |  |  |
| 1 | 0/2 | 0/ACNB | 16/13 | 0/0 | None |
| 2 | 0/0 | 0/0 | 21/14 | 0/0 | None |
| 3 | 0/0 | 0/0 | 14/14 | 0/0 | None |
| 4 | 0/0 | 0/0 | 14/16 | 0/0 | None |
| 5 | 0/0 | 0/ASP | 14/21 | 0/0 | None |
| 6 | 0/0 | 0/0 | 17/22 | 0/0 | None |
| 7 | 0/2 | 0/0 | 15/17 | 0/0 | Hypotension (COND) |
| 8 | 0/1 | 0/EC | 14/18 | 0/0 | None |
| 9 | 0/ 0 | 0/0 | 16/24 | 0/0 | Perianal abscess (COND) |
| 10 | 0/1 | 0/KPC | 14/17 | 0/0 | None |
| 11 | 0/0 | 0/GRAM(-) | 3/20 | 13/9 | Bradycardia (COND) |
| 12 | 2/0 | 0/0 | 16/15 | 0/0 | None |
| 13* | 0/0 | 0/0 | - | 0/0 | Granuloma ostomy (COND) |
| 14 | 0/0 | 0/0 | 3/12 | 0/0 | None |

Mob: mobilization; Cond: conditioning; ACNB: Acinetobacter; EC: *Escherichia coli*; ASP: Aspergillus; KPC: Klebsiella pneumoniae carbapenemase; GRAM(-): Gram Negative
