# Supplementary material for: Low toxicity and favorable clinical and quality of life impact after non-myeloablative autologous hematopoietic stem cell transplant in Crohn’s disease
Source: BMC Res Notes. 2017 Oct 6;10:495. doi: 10.1186/s13104-017-2824-1 (PMC5639601; doi:10.1186/s13104-017-2824-1)
Supplement: Supplementary file 3 — Additional file 3. Hematological abnormalities during autologous hematopoietic stem cell transplantation. [file 13104_2017_2824_MOESM3_ESM.docx]

**Hematological abnormalities during autologous hematopoietic stem cell transplantation** (mobilization/conditioning)

| Patient | Leucopenia  <1.0 x 10^9^/L | Granulocytopenia | Lymphotocytopenia  <0.5 x 10^9^/L | Thrombocytopenia  Platelets <25 x 10^9^/L | Anemia  Hb <10 g/dL | Neutropenia  <0.5 x 10^9^/L |
| --- | --- | --- | --- | --- | --- | --- |
| 1 | 1/8 | 1/7 | 1/7 | 0/0 | 4/12 | 0/7 |
| 2 | 7/7 | 5/7 | 4/7 | 0/0 | 11/11 | 2/6 |
| 3 | 4/8 | 3/7 | 3/7 | 0/0 | 0/13 | 2/6 |
| 4 | 7/12 | 7/10 | 7/11 | 0/0 | 7/14 | 1/8 |
| 5 | 4/11 | 4/10 | 3/10 | 0/0 | 8/15 | 1/9 |
| 6 | 7/11 | 4/7 | 3/16 | 1/10 | 8/17 | 3/7 |
| 7 | 8/12 | 7/11 | 7/13 | 0/6 | 8/11 | 4/8 |
| 8 | 4/13 | 6/12 | 6/17 | 0/10 | 0/14 | 2/9 |
| 9 | 0/12 | 1/10 | 9/14 | 0/5 | 0/14 | 0/7 |
| 10 | 3/13 | 2/12 | 3/17 | 0/15 | 0/11 | 1/11 |
| 11 | 3/15 | 3/10 | 4/18 | 0/8 | 10/18 | 0/8 |
| 12 | 4/9 | 4/8 | 9/11 | 0/5 | 9/15 | 4/7 |
| 13 | 5/10 | 4/8 | 10/13 | 0/10 | 10/15 | 4/8 |
| 14 | 0/8 | 0/7 | 1/15 | 0/5 | 3/15 | 0/6 |
